# Novel insights into relationships between metabolic covariance patterns of FDG-PET data and clinical status in Parkinson's disease using partial least squares correlation analysis

**DOI:** 10.1177/1877718X251394778

**Published:** 2025-11-11

**Authors:** Connor WJ Bevington, Sahib Dhaliwal, Jessamyn McKenzie, A Jon Stoessl, Vesna Sossi

**Affiliations:** 1 Department of Physics and Astronomy, 8166 University of British Columbia, Vancouver, BC, Canada; 2 Pacific Parkinson's Research Centre, 539641 University of British Columbia, Vancouver, BC, Canada; 3 Department of Medicine, Division of Neurology, 539222 University of British Columbia, Vancouver, BC, Canada; 4 215463 Djavad Mowafaghian Centre for Brain Health, University of British Columbia, Vancouver, BC, Canada

**Keywords:** metabolic covariance patterns, Parkinson's disease, healthy aging, positron emission tomography, metabolic rate of glucose, multivariate modeling

## Abstract

**Introduction:**

Metabolic covariance patterns derived from imaging data help characterize disease-related physiological changes in several neurodegenerative disorders, including Parkinson's disease (PD), but their relevance to different disease stages and/or clinical variables related to disease or disease predisposition, such as age, is often unclear.

**Methods:**

We incorporated clinical information in deriving metabolic covariance patterns relevant to different aspects of PD: disease initiation, disease progression, and physiological similarities in PD and healthy aging. This was achieved by combining Partial Least Squares Correlation analysis with Scaled Subprofile Modeling (SSM-PLSC).

**Results:**

When combining PD and HC data, SSM-PLSC identified a spatial pattern similar to the well-known PD-related disease pattern as expected; when applied to PD-only data—thus emphasizing disease progression—the spatial pattern became characterized by expanding putaminal hypermetabolism and reduced emphasis on cerebellar hypermetabolism. Finally, when applied to PD and HC data but permitting a different dependence on clinical variables, SSM-PLSC identified a spatial pattern with relative hypermetabolism in the basal ganglia, brain stem, and white matter together with relative hypometabolism in frontal cortex; in HC this pattern solely related to age, while in PD the same pattern significantly correlated with both age and disease duration.

**Conclusion:**

We identified metabolic patterns that are more closely related with different aspects of PD, directly derived from relationships between metabolic alterations and clinical variables. We also revealed metabolic signatures common to PD and aging, which may highlight age-related metabolic changes that form a predisposition to PD, as age is the single highest risk factor for PD.

## Plain language summary title

Brain changes in Parkinson's disease and their relationship to clinical aspects of disease

**Plain language summary:**

Previous research has identified patterns of changes to how the brain consumes glucose (sugar) in Parkinson's disease (PD), compared to healthy individuals of a similar age, which has an impact on how the brain generates and uses energy. However, PD is a complex disease that progresses over time, so it is important to understand how these patterns relate to different disease stages, disease severity, and to risk factors for developing PD—the most prominent being age. To achieve this, we analyzed brain imaging data of glucose in individuals with PD and healthy individuals with a technique that looks at the relationship between the imaging data and clinical information relevant to disease and age. This technique enabled us to observe how brain changes associated with having PD—that is, how the PD brain differs from the healthy brain—compared to changes that occur at different stages of disease. We found that some brain changes initially present in PD continue to worsen as disease becomes more severe, whereas others are unchanged. Additionally, by searching for common brain changes in PD and healthy individuals we identified brain regions that are affected only by aging in healthy individuals, but by aging *and* disease in PD. This suggests that these age-related brain changes may contribute to an increased likelihood of developing PD, since age is the highest risk factor for developing this disease. Collectively, the patterns identified in this work help increase our understanding of the specific brain changes that occur in the development and progression of PD.

## Introduction

Parkinson's disease is the second most common neurodegenerative disorder with increasing worldwide prevalence.^1^ While its origins are not completely understood, it is widely recognized that its biological underpinnings—some of which precede clinical symptoms by many years—are heterogeneous in nature.^2^ Likewise, some PD risk factors have been clearly identified, such as the occurrence of rapid eye movement sleep behaviour disorder (RBD)^3^ and—most notably—aging itself,^4^ both of which may be associated with different disease trajectories. However, the pathogenic mechanisms associated with these risk factors are still unknown, as are factors that lead to different disease manifestation and progression.

Neuroimaging is deemed an important tool in seeking explanations to these yet unanswered questions by providing in vivo imaging biomarkers, which may help characterize disease initiation, progression, and/or prognosis.^5^ In this context, metabolic imaging with [^18^F]fluorodeoxyglucose Positron Emission Tomography (FDG-PET) is considered one of the most promising approaches as it can provide insights into several disease- and treatment-related complications and is particularly helpful in separating PD from other synucleinopathies and neurodegenerative diseases. Key to this is the identification of the Parkinson's Disease-Related Pattern (PDRP), a metabolic spatial covariance pattern found to be highly specific for PD.^6,7^

The PDRP was first demonstrated using FDG data, characterized by relative hypermetabolism in pons, cerebellum, globus pallidus, and posterior putamen and relative hypometabolism in premotor and occipitoparietal regions. Instead of treating each region of the brain as independently modified by disease, the linear combination of regions forming the PDRP are collectively modified by disease—better reflecting the interconnectedness of brain function. The PDRP is robust to test–retest conditions,^7^ image reconstruction method,^8^ and ethnicity of the cohort.^9–11^ Importantly, its subject-specific “expression” also correlates with clinical variables of motor impairment^6,12,13^ and is altered by therapeutic intervention.^7,^^14–16^

While the PDRP relates to overall motor function in PD, other covariance patterns are more closely related to tremor^17^ and to cognitive impairment.^18^ The PDRP is thus suboptimally suited to capture the metabolic signatures associated with the full range of disease presentations, since it does not directly investigate the correlations between specific metabolic alterations and subject status, i.e., clinical metrics across multiple disease domains and PD-related risk factors, such as age. Such knowledge would help unravel the impact of individual clinical characteristics on brain metabolic patterns and potentially identify clinical aspects that synergistically contribute to specific brain alterations.

The goal of this work is thus to investigate the existence of patterns of metabolic alterations that relate to specific aspects of PD—namely (i) disease initiation, (ii) disease severity/progression, and (iii) potential physiological similarities to aging and PD. To achieve this, we use a combination of Scaled Subprofile Modeling^19^ and Partial Least Squares Correlation analysis (**SSM-PLSC**), which provides a framework to *directly* explore the relationships between metabolic imaging data and a set of clinical variables. In addition to standard disease progression tracking metrics, we include age and a clinical evaluation of RBD severity in the set of clinical variables to query their possible contribution to metabolic alterations. Age is particularly relevant, given that aging is associated with many PD-related pathophysiological changes and may set the stage for more specific pathology,^20^ and that PD shows disruptions to the functional signatures of healthy aging.^21,22^

In this paper, we present three spatial patterns identified from combinations of PD and/or healthy control (HC) metabolic imaging data and corresponding clinical variables, each characterizing metabolic changes related to different aspects of PD reflecting: (i) metabolism differences between PD and HC (expected to closely resemble the PDRP obtained with SSM-PCA alone); (ii) metabolic changes specific to disease severity/progression; (iii) metabolic changes common between aging in HC and disease and aging in PD.

This paper proceeds as follows. First, a brief theoretical explanation of SSM-PLSC is provided as relevant to the interpretation of the results; precise mathematical details can be found in the Supplementary Information. Data acquisition, data preparation, and SSM-PLSC analysis is then described in detail. Next, the metabolism patterns listed above are presented and contrasted. Finally, the clinical and biological relevance of these patterns is discussed, and we finish by briefly identify how the present analysis could be extended to other PD subtypes and/or neurodegenerative disorders.

## Materials & methods

### SSM-PLSC: theory

SSM-PLSC seeks to identify paired spatial patterns and clinical patterns whose subject-specific “expressions” maximally covary. A spatial pattern is characterized by a weight in each brain region which signifies relative increases or decreases in the chosen imaging metric; in the present application, this corresponds to relative hypermetabolism or hypometabolism. The subject-specific “expression”, or subject score, is essentially a measure of the overlap between a given subject's imaging data and the spatial pattern. Likewise, a clinical pattern is characterized by weights for each clinical variable and its subject scores now indicate the overlap between a given subject's clinical status and the clinical pattern.

Since the subject scores for the spatial and clinical patterns maximally covary in SSM-PLSC, the clinical pattern subject scores help interpret the relationship between an individual's state (e.g., someone older but with a short disease duration vs. younger but with a longer disease duration) and the expression of functional brain alterations. This also implies the clinical variable weights are closely related to the correlation strength between the spatial pattern subject scores and each clinical variable. As a result, SSM-PLSC provides a framework to *directly* explore the relationships between brain function and the clinical characteristics under consideration.

**Figure 1.**
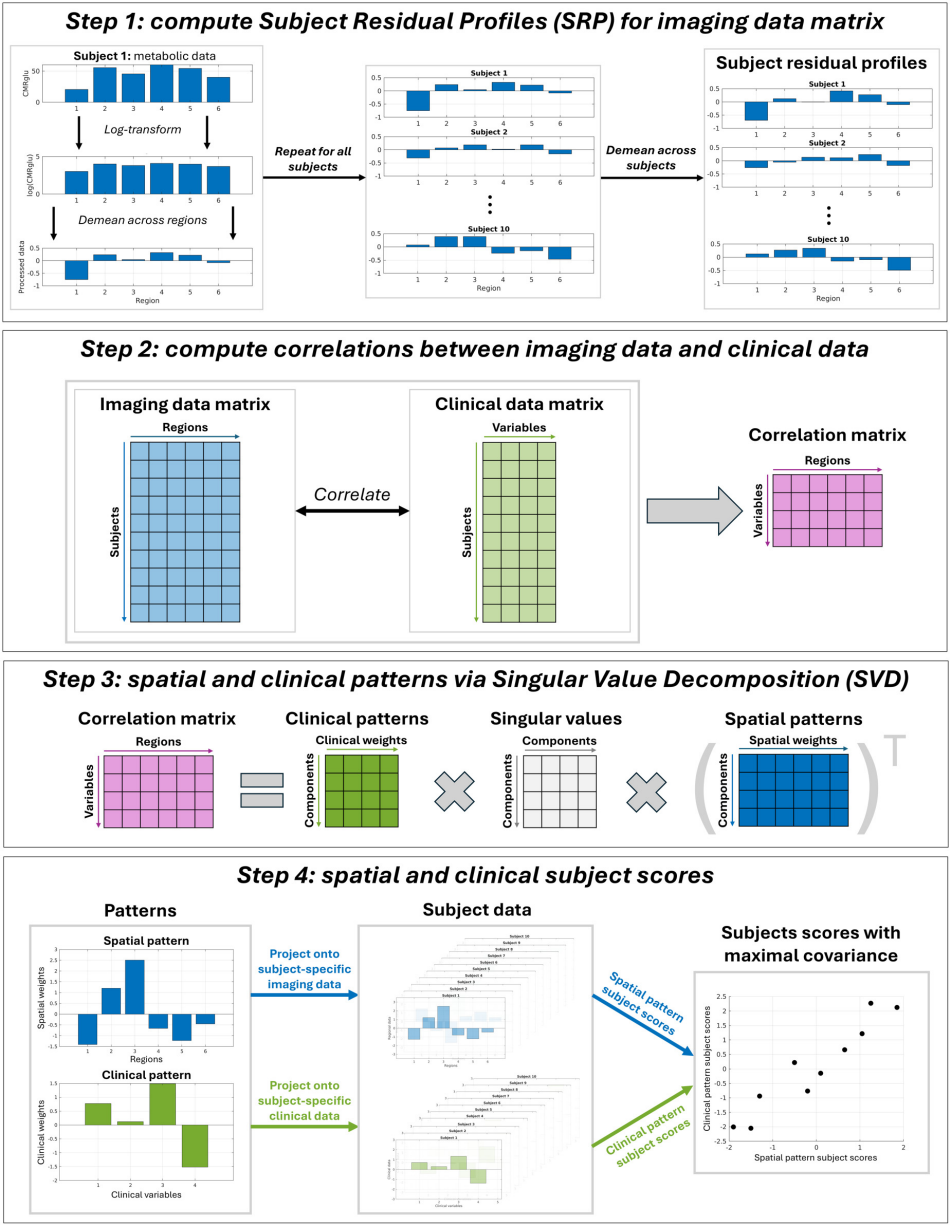
Illustrating the entire SSM-PLSC pipeline, including data pre-processing before spatial/clinical patterns and their associated subject scores are obtained.

To identify these paired spatial and clinical patterns, termed *components*, the data processing step of Scaled Subprofile Modeling (SSM^19^) is first applied to demean the log-transformed imaging data across subjects and regions, in order to yield a subject residual profile (SRP) that focuses on the biologically relevant metabolic variance across subjects. Partial Least Squares Correlation analysis (PLSC^23^) is then applied to the SRP, which first computes the correlations between each regional value and each clinical variable, forming a correlation matrix. The correlation matrix is finally decomposed using Singular Value Decomposition (SVD) into sets of paired spatial and clinical patterns. Subject scores are obtained by projecting the processed imaging and clinical data onto their respective patterns. Figure 1 illustrates the entire SSM-PLSC pipeline.

### Participants and demographics

21 individuals with PD and 21 age-matched HC were recruited as a part of a PET/MR study investigating brain energetics^24^ approved by the UBC Clinical Ethics Review Board under Application Nos. H19-01839 and H19-02892, following the ethical principles of the Declaration of Helsinki. Participants were excluded from participation if they had any chronic diseases (other than PD in the PD group) or if they had any indications preventing them from being scanned in a 3T MRI environment. HC were also excluded if they had an immediate family history of neurodegenerative disorders.

All participants underwent assessments of motor performance (Movement Disorder Society-Unified Parkinson's Disease Rating Scale Part III [UPDRS-III]^25^), and sleep behavior (REM Behaviour Disorder Screening Questionnaire [RBDSQ]) and provided demographic information before scanning, which serve as quantitative clinical metrics for PLSC. All clinical evaluations and FDG PET for the PD group were performed in the OFF medication state (withdrawn from levodopa for at least 12 h). For PD, the more affected side was identified by summing over bilateral UPDRS-III items separately for the left and right side. Table 1 summarizes the clinical data, which were used as input to SSM-PLSC. As expected, the PD group had significantly higher UPDRS-III and RBDSQ compared to HC.

**Table 1.** Clinical data for PD and HC participants.

Metric	HC (n = 21)			PD (n = 21)			p (PD-HC)	Effect size d (PD-HC)
	Mean ± std	IQR	[min, max]	Mean ± std	IQR	[min, max]		
Age	62.7 ± 9.7	[58, 67]	[42, 80]	65.2 ± 6.9	[60, 68]	[50, 76]	0.34	0.19
Disease duration	-	-	-	7.1 ± 4.1	[4, 8]	[2, 18]	-	-
UPDRS-III	4.8 ± 2.3	[3, 6]	[1, 9]	23.6 ± 7.1	[17, 26]	[16, 43]	**< 10^−13^**	3.5
RBDSQ	1.6 ± 1.4	[0, 3]	[0, 4]	5.2 ± 2.5	[4, 7]	[2, 11]	**< 10^−5^**	1.6

UPDRS-III: Unified Parkinson's Disease Rating Scale Part III; RBDSQ: REM Behaviour Disorder Screening Questionnaire; std: standard deviation; IQR: interquartile range. P-values were calculated from a two-sample, two-tailed t-test. Effect size was computed using Cohen's d. Bold indicates a significant difference between PD and HC (p<0.05).

### Imaging data acquisition, processing, and analysis

As part of the overall imaging protocol, subjects underwent a dynamic 60-min FDG scanning session on the GE SIGNA PET/MR to obtain quantitative parametric maps of cerebral metabolic rate of glucose (CMR_glu_). A T1-weighted MPRAGE sequence (TR/TE/TI = 7.688/3.12/900 ms, flip angle 8°, 1 mm isotropic voxels, 202 slices) was acquired simultaneous with FDG to provide an anatomical reference.

The FDG data were grouped into 4 × 1, 3 × 2, 8 × 5, 1 × 10 min time frames and reconstructed using PSF-HYPR4D-K-TOFOSEM.^26^ Rigid frame-to-frame realignment was performed using SPM12 to account for any motion during the scan. Voxelwise maps of CMR_glu_ were computed as:

(1) $$CMR_{glu}=K_{i}\times \frac{C_{glu}}{LC}$$

where $K_{i}$ is the net FDG uptake constant obtained using the Patlak method^27^ with an image-derived carotid input function,^26^ LC=0.65 is the “lumped constant” accounting for differences in phosphorylation between glucose and FDG, and $C_{glu}$ is the plasma glucose concentration obtained from blood samples $C_{glu}$ values were obtained from a blood sample prior to injection and a second blood sample 40 minutes post-injection to monitor any variation during scanning; these values were averaged to provide a single value for $CMR_{glu}$ calculations. In any case, the subject-specifc $C_{glu}$ value and the global LC value do not factor in the SRP used for pattern derivation; see the Appendix for a derivation. The CMR_glu_ images were co-registered to each participant's T1-weighted image using FSL.

### SSM-PLSC: data preparation and analysis

Due to the relatively modest number of subjects in our dataset, we used a regional approach for pattern analysis. A segmentation of the T1-weighted image into cerebral white matter, cerebellar grey/white matter, cortical, and subcortical regions of interest (ROI) was obtained using Freesurfer.^28^ Since progressive dopaminergic dysfunction in PD tends to result in anterior–posterior pathophysiological and microstructrual gradients in the putamen,^29–33^ we manually subdivided the putamenal ROIs into thirds along this axis. Furthermore, we manually divided the Freesurfer brainstem ROI into separate ROIs for the pons, medulla, midbrain, and bilateral substantia nigra, drawn by an expert neurologist. These modifications resulted in a total of 97 ROIs for analysis, which are listed in the Supplementary Information. Data from the PD participants were flipped as necessary such that the left side of the brain was the more affected side. Data from the HC participants were not flipped.

We applied SSM-PLSC in three separate ways to identify patterns related to different aspects of PD:

- **PD vs. HC patterns:** first, we combined PD and HC data in the SSM-PLSC analysis to obtain patterns that should reflect primarily the **metabolism differences in PD vs. HC**. This was essentially a method consistency/validation step as we expected this pattern to be very similar to the PDRP.
- **PD-only patterns:** we then applied SSM-PLSC to PD-only data to focus on variance *within* PD, thus yielding **patterns tracking metabolism variations within PD** most strongly related to the clinical metrics used.
- **PD and HC patterns:** finally, we jointly analyzed separately computed SRPs for PD and HC and within the PLSC framework, ultimately producing **common metabolism patterns with unique clinical expression in PD and HC**, i.e., a single spatial pattern but separate clinical patterns for each group. This approach is best suited to identify metabolism changes in healthy aging that are also present in PD.

### Pattern validation and statistical testing

A random permutation test to assess pattern generalizability (10,000 permutations, p < 0.05) was performed by computing correlation matrices between the imaging and clinical data after randomly shuffling subjects within the imaging data matrix while leaving the clinical data matrix untouched. Applying SVD to these permuted correlation matrices allows us to test the null hypothesis of the singular values from our derived components; a component that overfits to the subject sample will have a singular value less separated from the null distribution of singular values (i.e., a higher p-value) and thus has a reduced likelihood of generalizing to the population. Additionally, bootstrap resampling to assess the stability of the regional weights in each spatial pattern (10,000 bootstraps) was performed. Precise computational details of these validation techniques can be found in reference.^23^ Software packages for both SSM (https://feinsteinneuroscience.org/imaging-software/download-software) and PLSC (https://www.rotman-baycrest.on.ca/index.php?section = 84) are freely available online; for the purposes of combining these two methods we wrote in-house MATLAB scripts based on the algorithmic details in references.^7,23^ This code will be made freely available upon request.

As in previous SSM-PCA analyses, we assessed whether the spatial subject scores in the PD vs. HC patterns significantly separate the two groups using a two-tailed t-test. In contrast, the spatial subject scores in the PD and HC patterns cannot be meaningfully compared since these scores are derived from separate PD and HC SRPs, meaning the subject scores capture intra-group variance in pattern expression.

We also explored PD vs. HC differences in the SRPs (i.e., before being entered into PLSC) in regions where the spatial weights have noteworthy differences between patterns. A two-tailed t-test was used as we have no a priori assumption on the direction of difference. P-values provided from these tests are uncorrected, as these comparisons serve to contextualize how key region-wise differences contribute to the overall spatial patterns and should not be taken as formal statistical tests of group differences within specific regions. Likewise, since our SSM-PLSC patterns are derived from correlations between SRP values and clinical variables, we also provided Pearson's correlation coefficients and uncorrected p-values for these relationships in these same regions.

## Results

### Metabolism differences in PD vs. HC

**Figure 2.**
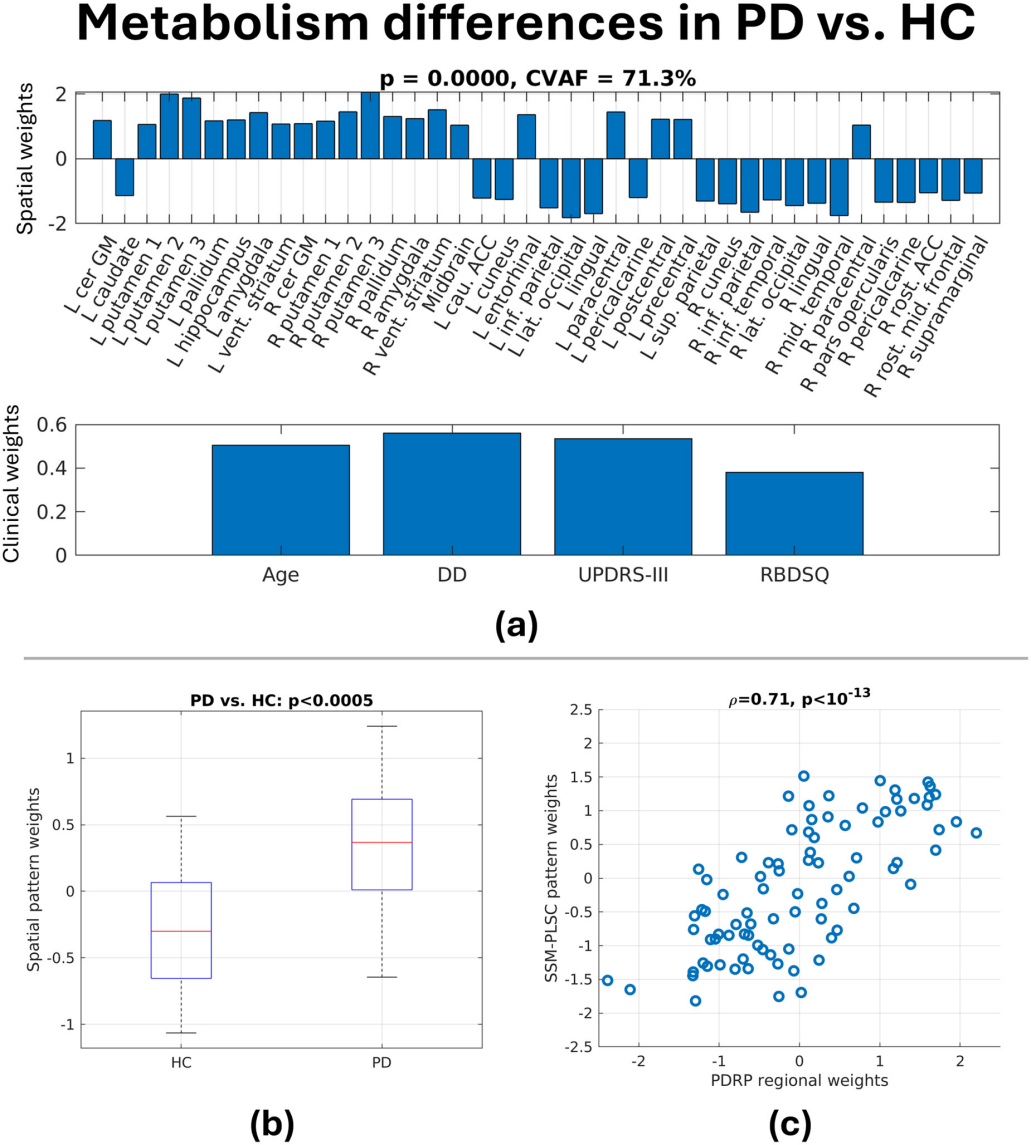
(a) The first spatial pattern (top pane) and clinical pattern (bottom pane) derived from combined HC and PD data. In (b), the spatial subject scores are shown to significantly separate PD vs. HC. In (c), the spatial weights are shown to resemble those from the previously derived Parkinson’s Disease Related Pattern (PDRP).^7^ CVAF = percentage covariance accounted for by the pattern set; DD = disease duration; UPDRS-III = Unified Parkinson’s Disease Rating Scale Part III; RBDSQ = REM sleep Behaviour Disorder Screening Questionnaire.

SSM-PLSC identified two significant components (p < 0.0001 and p < 0.05, respectively). The first component is presented in Figure 2(a); for spatial pattern weights (top pane), positive values indicate relative hypermetabolism and negative values indicate relative hypometabolism. Spatial weights have been z-scored and only regions with |z|>1 and stable after bootstrap resampling are shown to retain the most important regions in the pattern. This spatial pattern consists of PDRP-like regional weights, characterized by relative hypermetabolism in cerebellum, putamen, pallidum, pre/postcentral gyri, and paracentral lobule, and widespread cortical relative hypometabolism, primarily in occipitoparietal regions.

The corresponding clinical pattern is shown in the bottom pane of Figure 2(a); for clinical weights, dark shading indicates the 95% confidence interval of the correlation coefficient between spatial subject scores and the corresponding clinical variable does not cross zero. This clinical pattern has significant positive weighting for all clinical variables, meaning spatial pattern expression is associated with the combination of increasing age, disease duration, UPDRS-III, and RBDSQ. Since HC and PD are grouped together, disease duration, UPDRS-III, and RBDSQ function partially as discriminatory variables between HC and PD. As a result, the spatial subject scores separate PD vs. HC (Figure 2(b), p < 0.0005, one-tailed, two-sample t-test). Additionally, the regional weights correlate to regional averages of a voxelwise PDRP^7^ (Figure 2(c)).

The second significant SSM-PLSC component is presented in Supplementary Figure S1. The associated clinical pattern has positive weighting for age but negligible weighting for disease duration and UPDRS-III. Since the primary component captures essentially all PD vs. HC covariance, this secondary component appears to capture common intragroup covariance related to aging that is independent of disease.

### Patterns that track metabolism variations within PD

**Figure 3.**
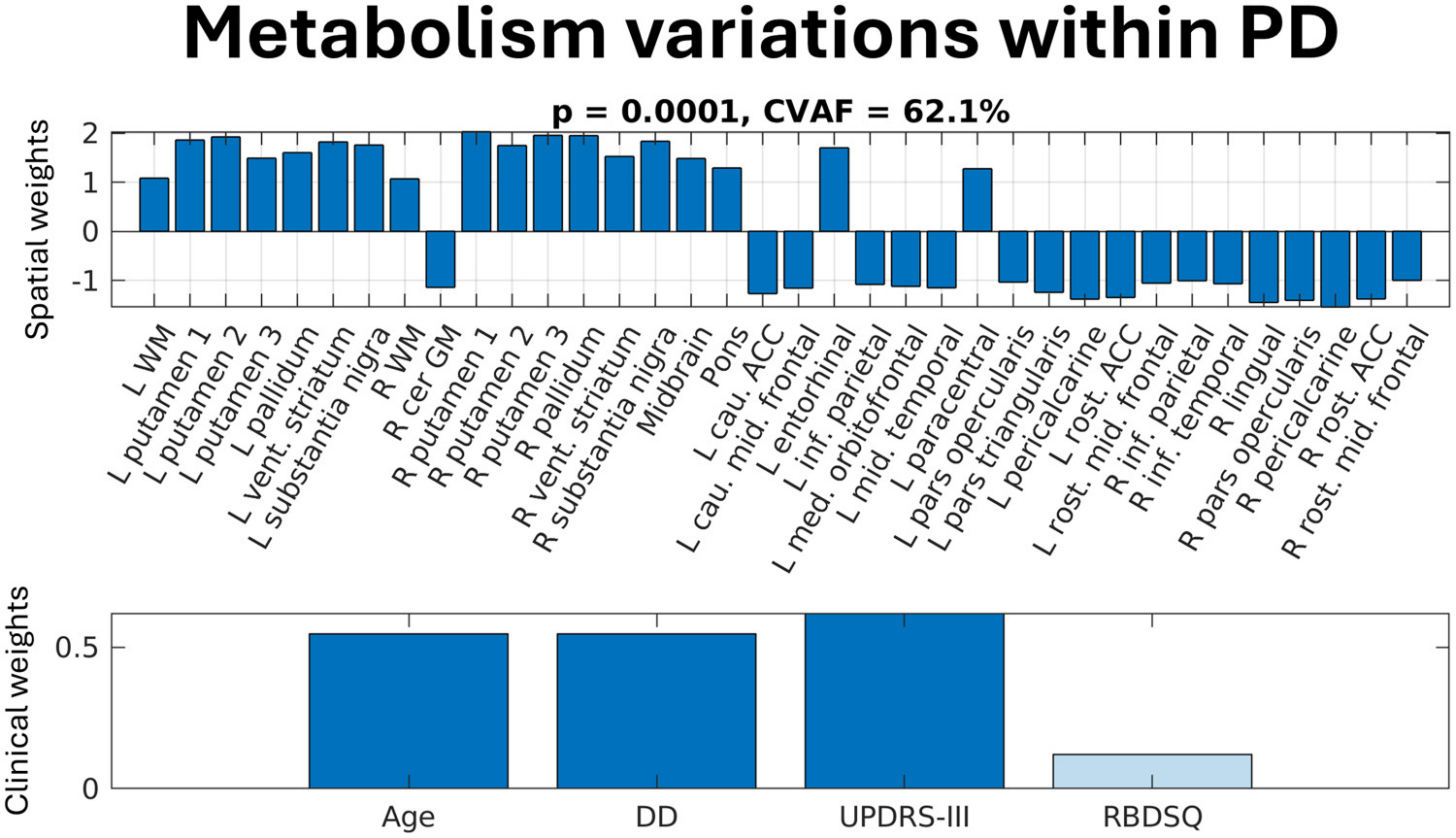
The spatial pattern (top pane) and clinical pattern (bottom pane) derived from PD-only data. CVAF = percentage covariance accounted for by the pattern set; DD = disease duration; UPDRS-III = Unified Parkinson’s Disease Rating Scale Part III; RBDSQ = REM sleep Behaviour Disorder Screening Questionnaire.

Restricting SSM-PLSC to the PD group results in a single significant spatial pattern (p < 0.001, Figure 3(a)) with similar regions of relative hyper/hypometabolism as the first PD vs. HC pattern of Figure 2(a). The corresponding clinical pattern has significant positive weightings for age, disease duration, and UPDRS-III, but not for RBDSQ. This indicates that these PD-related functional alterations tend to scale with motor symptom severity rather than RBD symptomology.

**Figure 4.**
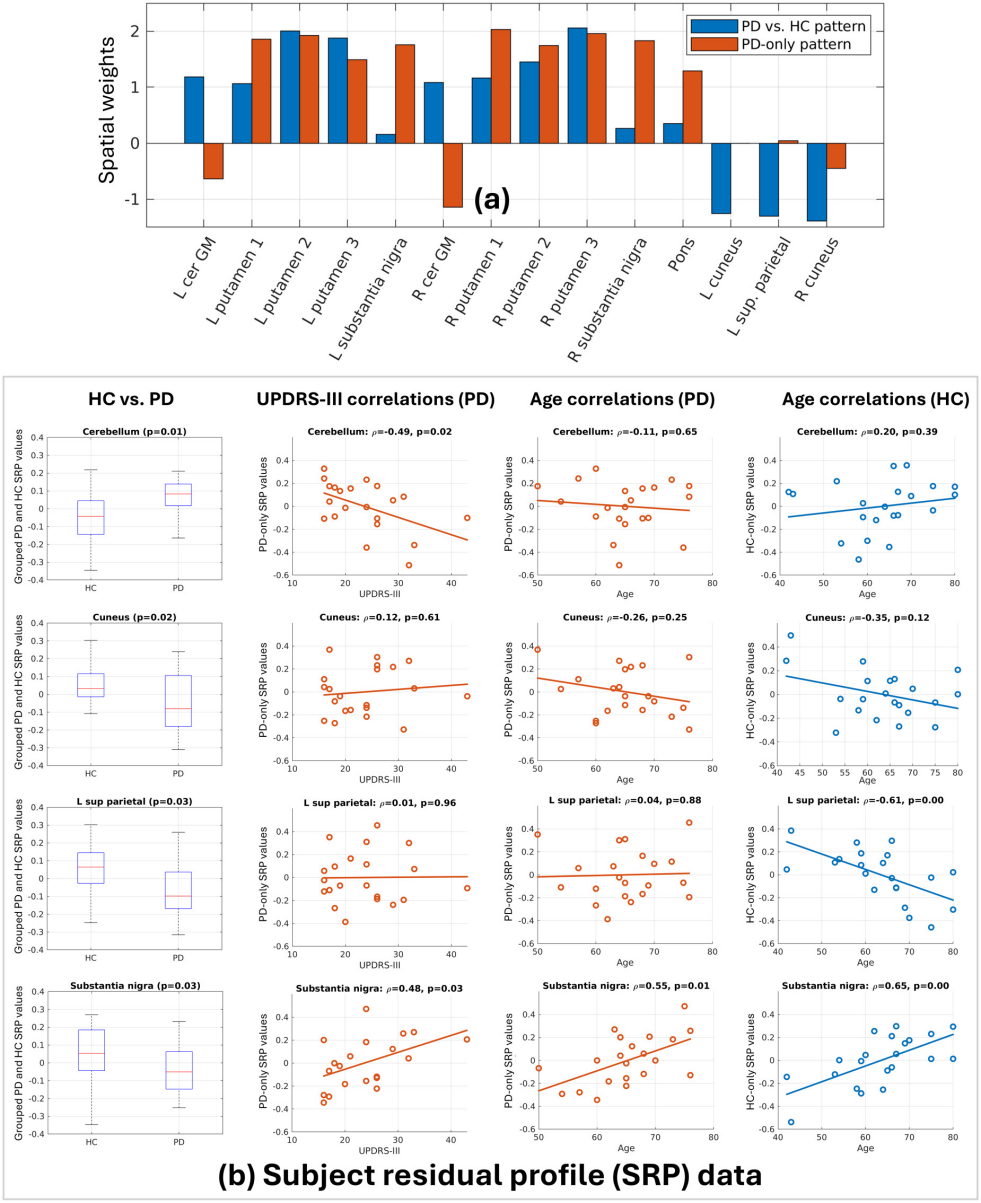
(a) Highlighting the differences in spatial weights between the PD vs. HC pattern of Figure 2a and PD-only pattern of Figure 3. (b) In representative regions we showcase: values from the grouped PD and HC SRP (leftmost column); correlations between UPDRS-III and PD-only SRP values (left-centre column); correlations between age and PD-only SRP values (right-centre column); correlations between age and HC-only SRP values (rightmost column).

Despite spatial similarity to the PD vs. HC pattern, subtle yet key differences in the specific regional weights exist, which are contrasted in Figure 4(a). To help explain these differences, values from the SRPs—i.e., relative metabolic data obtained by demeaning log-transformed regional data across regions then subjects—used to derive these patterns are provided in Figure 4(b). Within this subplot, the leftmost column compares SRP values derived from grouped PD and HC data in representative regions; group differences here are most important for determining spatial weights in the PD vs. HC pattern, since the PD-related clinical variables act partially as discriminatory variables. The second column visualizes correlations between values of the PD-only SRP and UPDRS-III; strong relationships here will contribute to spatial weights in both the PD vs. HC and PD-only patterns. The final two columns assess age relationships with PD-only HC-only SRP values, respectively, which provides context on whether strong spatial weights in the patterns are partially the result of associations with age, disease, or both.

Most notably, the PD vs. HC pattern includes stronger weightings than the PD-only pattern in cerebellum, cuneus, and superior parietal cortex; in these regions relative metabolism differentiates PD vs. HC but changes minimally with increasing disease severity. Conversely, substantia nigra has high weighting in the PD-only pattern but essentially no weighting in the PD vs. HC pattern. Finally, the relative hypermetabolism across the putamen subdivisions differs in these patterns: the PD vs. HC pattern shows greater weighting in bilateral posterior putamen, whereas the PD-only pattern has high weighting across the putamen (albeit slightly higher in anterior putamen). These weightings mirror progressive dopaminergic losses in PD, with losses typically confined to the more affected posterior putamen in early disease before progressing more anterior and eventually bilaterally. Therefore, this PD-only analysis with SSM-PLSC focuses on the functional alterations associated with increasing disease severity, found not to be identical to the set of functional alterations that discriminate PD vs. HC.

### Common metabolism patterns with unique clinical expression in PD and HC

**Figure 5.**
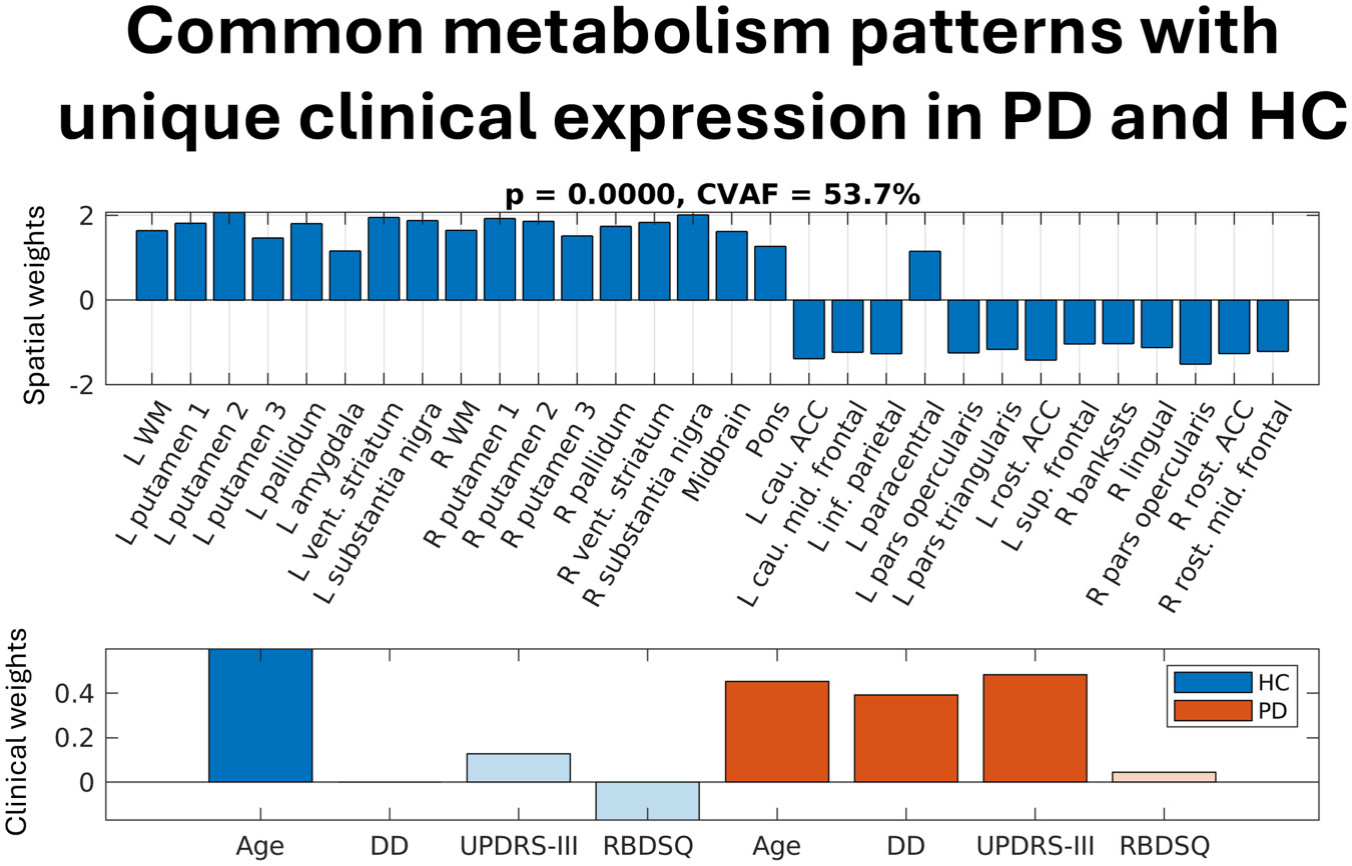
The spatial pattern (top pane) and clinical pattern (bottom pane) derived from separately processed PD and HC data. CVAF = percentage covariance accounted for by the pattern set; DD = disease duration; UPDRS-III = Unified Parkinson’s Disease Rating Scale Part III; RBDSQ = REM sleep Behaviour Disorder Screening Questionnaire.

Grouping HC and PD separately results in a single common spatial pattern (p < 0.0001, Figure 5(a)) characterized by hypermetabolism in cerebral white matter, basal ganglia, and brain stem substructures, as well as hypometabolism in anterior cingulate cortex and frontal cortical regions. In PD, spatial pattern expression is associated with the combination of increasing age, disease duration, and UPDRS-III in PD. In HC, spatial pattern expression is only associated with increasing age.

**Figure 6.**
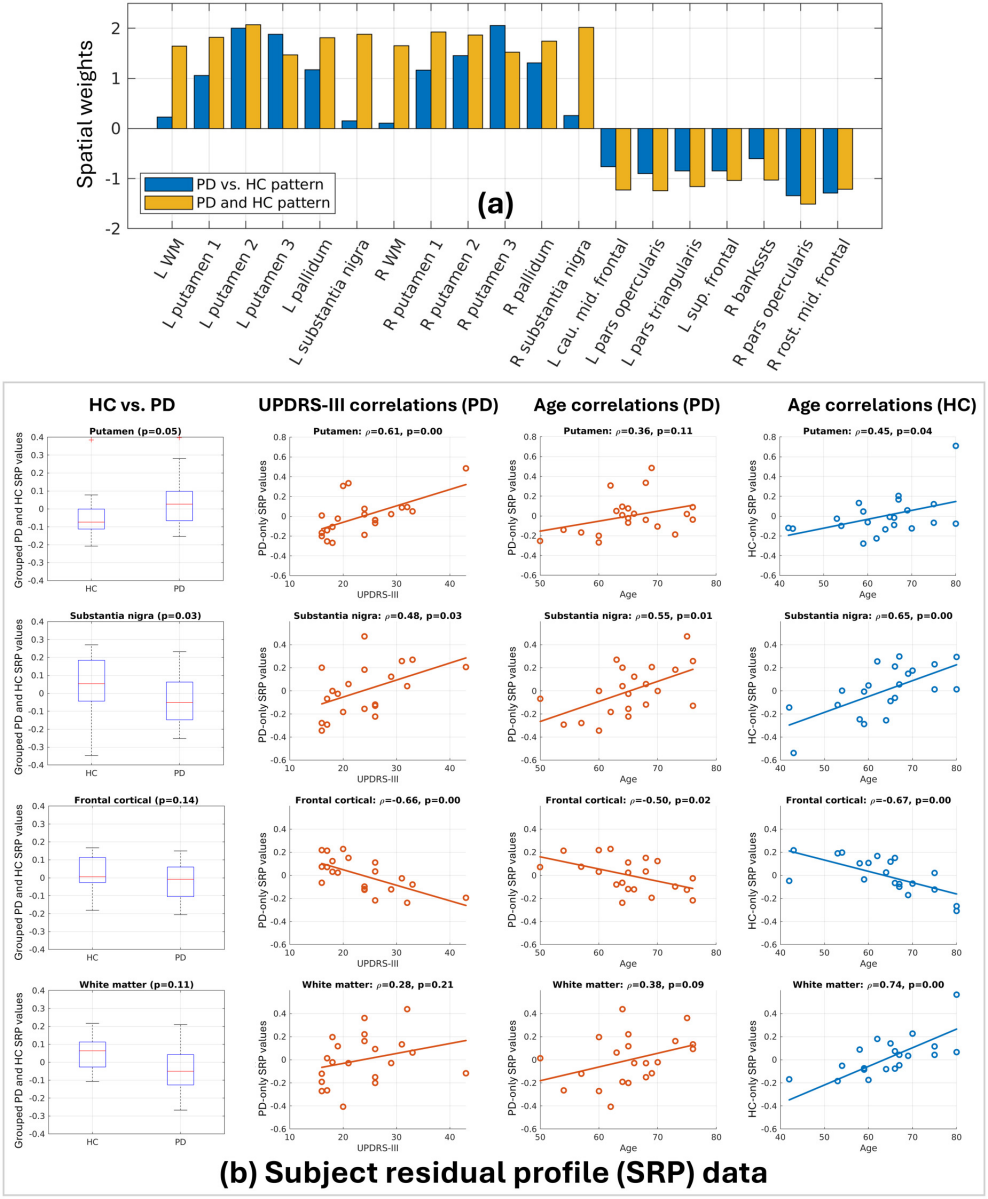
(a) Highlighting the differences in spatial weights between the PD vs. HC pattern of Figure 2a and the PD and HC pattern of Figure 5. (b) In representative regions we showcase: values from the grouped PD and HC SRP (leftmost column); correlations between UPDRS-III and PD-only SRP values (left-centre column); correlations between age and PD-only SRP values (right-centre column); correlations between age and HC-only SRP values (rightmost column). Frontal cortical indicates the averaged values across the rightmost seven ROIs in (a).

With HC and PD grouped separately, this identifies a pattern of metabolism changes associated with aging that are potentially influenced by disease. Figure 6(a) contrasts the weightings of this PD and HC pattern with the PD vs. HC pattern of Figure 2(a) and identifies regions such as putamen, substantia nigra, and frontal cortical regions where intragroup correlations reveal relative metabolism changes with age in both HC and PD, but in PD these changes are further modulated by UPDRS-III (Figure 6(b)). In general, the variance explained in relative metabolism by age is lower in PD—even in regions where there is no observed correlation with disease metrics, such as cerebral white matter—indicating either a disruption to aging by disease or a floor effect in PD where age no longer has any further impact.

## Discussion

In this work, we specifically characterize the metabolic alterations associated with different aspects of PD by leveraging the combination of SSM-PLSC pattern analysis. The combination of metabolic data and clinical metrics allowed us to identify: (i) metabolism patterns that distinguish PD and HC (PD vs. HC pattern, Figure 2); (ii) patterns that track metabolism variations within PD only (PD-only pattern, Figure 3); and (iii) metabolism patterns common to both PD and HC that have unique clinical expression (PD and HC pattern, Figure 5). By using age as a variable alongside traditional PD-related clinical metrics, these patterns collectively offer novel insights into the metabolic signatures related to disease initiation, severity/progression, and similarities to the healthy aging process.

While a comprehensive tutorial has demonstrated the utility of PLSC in neuroimaging data,^23^ at the time of writing there are only two published studies in the PD literature where the relationship between PET imaging and external information is studied using PLSC (to the best of our knowledge); one investigated relationships between combined FDG and resting-state fMRI data to gene expression data^34^ and the other probed relationships between FDG data and clinical variables of visual illusions.^35^ A unique “flavour” of Partial Least Squares that investigates causal models has also been used to test different neurodegeneration progression models in PD disease subtypes using multimodal MRI data,^36^ and relationships between cortical atrophy and mitochondrial gene expression in prodromal synucleinopathies have also been probed by PLS.^37^

### Improved clinical context for PD-related metabolism alterations

Previous pattern analyses, using only metabolic data, have identified a robust PD-related disease pattern (PDRP) that is optimized to differentiate PD from HC.^6,7^ Univariate, post-hoc correlations demonstrate that PDRP expression relates to disease duration and/or UPDRS-III, indicating that the PDRP captures a set of metabolic changes associated with disease initiation that tends to scale with disease severity. By including metabolic data and clinical variables in our SSM-PLSC analysis, we directly explore the relationships between metabolism and clinical status to produce a PD vs. HC pattern whose expression also separates PD from HC (Figure 2(b)), whose spatial weights are similar to those of the PDRP (Figure 2(c)), and—mostly importantly—demonstrate increasing expression with a combination of disease duration, UPDRS-III, and age (Figure 2(a)).

The linear combination of these variables forms the clinical subject scores, which can be thought of as one “mega-variable” characterizing individualized clinical status, as related to the metabolic alterations present in this pattern. This explains how these metabolic changes distinguish HC vs. PD: HC have no disease duration and low UPDRS-III and RBDSQ scores leading to lower clinical subject scores compared to PD and hence significantly reduced expression of the metabolic changes. Context can also be provided for within-group variability of these metabolic changes. For instance, given two individuals with PD having similar age and UPDRS-III but one having a significantly longer disease duration, the overall expression of the metabolic changes will be stronger in the individual with the longer disease duration—despite their apparent better compensation. Alternatively, if a younger and an older subject are both newly diagnosed with PD and have relatively low UPDRS-III, the older individual will have a higher pattern expression. Likewise, within HC age largely determines clinical subject scores, meaning the expression of these metabolic changes is relatively higher for older individuals, though as a group HC do not express these changes as strongly as PD (Figure 2(b)). This can be interpreted as a disruption of the normal aging process—a conclusion shared from a less direct investigation in reference.^21^

### Disease progression patterns vs. disease-related patterns

As the clinical manifestations of PD vary and/or progress it is reasonable to assume associated variations in metabolic alterations. This can be queried by applying SSM-PLSC analysis to PD-only data, although ideally the analysis should be applied to longitudinal rather than cross-sectional data; the resulting PD-only spatial pattern (Figure 3(a)) reveals several differences from the PD vs. HC pattern (contrasted in Figure 4(a)). For instance, in the PD vs. HC pattern relative hypermetabolism is more prominent towards the posterior end of the putamen—indicating this metabolic alteration best distinguishes PD vs. HC—whereas in the PD-only pattern the relative metabolism is similarly elevated across the putamen—indicating an expansion of relative hypermetabolism in the putamen is associated with increased disease severity. Relatedly, regions such as cerebellum, cuneus, and more affected superior parietal cortex are present in the PD vs. HC pattern since their relative metabolism differentiates PD from HC (Figure 4(b)), but these regions are not highly weighted in the PD-only pattern due to limited associations between relative metabolism changes and disease severity and/or age. These subtle differences between spatial patterns provide insights into the metabolic alterations that may be shared or unique between disease initiation and increased disease severity.

The complementary clinical patterns share similar weightings for age, disease duration, and UPDRS-III, but not for RBDSQ: the PD vs. HC pattern has a significant weight whereas the PD-only pattern does not. This is primarily a statistical artifact, since combining PD and HC results in RBDSQ acting a discriminatory variable for PD (Table 1). This lack of relationship agrees with previous work where SSM-PCA patterns related to idiopathic RBD are positively expressed in PD groups, but not differentially between PD groups with and without RBD symptoms.^38,39^

### Common spatial patterns with different intragroup clinical relationships: aging disrupted by PD

A unique application of SSM-PLSC is identifying common spatial patterns between multiple groups where the relationship to clinical variables may be different. Applying SSM-PLSC to HC and PD as separate groups and including age and clinical variables related to PD effects identifies a spatial pattern that is strongly associated with aging in the HC group, whereas in the PD group associations withaging and disease severity are observed (Figure 5(a)). Age has been robustly demonstrated to have a significant effect on cerebral metabolism,^40–42^ but is also a major risk factor for PD^4^ and the pathophysiology of PD can be partially described as a form of advanced aging.^20^ However, previous attempts to investigate metabolic signatures of aging that are disrupted by PD have generally been indirect. Two previous studies have visually compared separately derived aging- and PD-related spatial covariance patterns.^22,40^ A slightly more direct analysis applied SSM-PCA to grouped HC and PD data and combined components to best predict age in HC.^40^ Relatedly, another study computed the difference between biological age in PD and an age predicted by the expression of an aging-related pattern.^21^

Our SSM-PLSC analysis *directly* uses age-related metabolism changes from HC data as well as age- and disease-related metabolism changes from PD data to derive a single spatial pattern common to HC and PD, thus highlighting the potential metabolic similarities and/or disruptions to healthy aging in PD (Figure 5(a)). Contrasting this PD and HC pattern with the PD vs. HC pattern (Figure 6) identifies the substantia nigra (SN) as a region with changes related to both age and disease: PD has lower relative metabolism than HC on a group level, but within each group positive relationships with age are observed, and an additional positive relationship with UPDRS-III is observed in PD. This suggests disease initiation is associated with relative hypometabolism in SN, but PD does not change the relative hypermetabolism that occurs with aging.

Conversely, in the putamen increasing relative hypermetabolism is associated with increases in both age and disease severity, suggesting an acceleration of the healthy aging process. Similarly, relative hypometabolism in frontal cortical regions (primarily middle and inferior frontal cortex) has similar relationships with both age and disease severity.

This analysis also highlights regions where the relationship between metabolism and age is disrupted rather than accelerated. For instance, relative hypermetabolism in white matter is observed with age in HC, but this relationship is weakened in PD. Additionally, no significant relationship with motor disease severity is observed in PD, perhaps signaling a disruption due to non-motor disease effects. Similarly, hypometabolism in the more affected superior parietal cortex is associated with age, but only within HC. No significant relationship with disease severity is observed in PD, though as a group this region is already relatively hypometabolic, likely indicating a floor effect where prodromal or early disease effects have already reduced metabolism and thus aging has no further effect in PD.

### Pattern interpretations: symptomatic and biological perspectives

The hallmarks of PDRP recovered in the SSM-PLSC patterns are basal ganglia hypermetabolism and occipitoparietal hypometabolism. The former is hypothesized to be related to the diminution in inhibitory neurocircuits as a result of dopaminergic cell loss in the basal ganglia, thus leading to hyperactivity,^43,44^ whereas the latter is more poorly understood, possibly explained by any combination of cortical pathology,^45^ impairment of cholinergic projections from the basal forebrain,^46–48^ or relatively reduced microglial activity in some regions.^49^ The shift of putamen hypermetabolism from primarily posterior to the remainder of the putamen in our PD-only SSM-PLSC pattern (Figure 4) agrees with the well-known spreading of dopaminergic denervation along this axis.^29–33^ The progressive hypometabolism in cortical regions observed as a function of disease severity may be due to extensive synaptic density reductions with increasing disease duration, linked to axonal damage and increasing alpha-synuclein burden,^50^ and is likely associated with reduced striatal-cortical connectivity stemming from basal ganglia dopaminergic cell loss early in disease.^51^ Progressive hypometabolism in frontal regions may be linked to altered cortico-striatal-thalamo-cortical function,^45^ whereas progressive hypometabolism in occipital-parieto-temporal regions may stem from progression of the aforementioned basal forebrain disease.^47,48^

In the PD and HC pattern (Figure 5), basal ganglia relative hypermetabolism was found to be a feature common to PD progression and healthy aging. This finding agrees with previous work that visually compared separately derived aging- and PD-related spatial covariance patterns.^40^ However, given unclear support for declining synaptic density as a function of age,^52,53^ the increase in relative metabolism may instead signal a regionally selective shift to less efficient cellular bioenergetics pathways in the aging brain, such as aerobic glycolysis,^24,54^ or, like PD, a diminishment of inhibitory controls in basal ganglia motor circuits, though due to unique pathophysiology.^55^ Absolute grey matter metabolism is known to decrease with age,^56–58^ so the observed relative hypermetabolism in white matter may merely indicate a slower decline in white matter relative to grey matter, or again may be related to shifts in bioenergetic pathways. Relatedly, relative hypometabolism in frontal regions with increasing age suggests that these regions experience increasing hypometabolism at a faster rate than the global brain average.

### Limitations and future applications

The results of this study are limited by the relatively low number of subjects; more subjects would allow for a better sampling of a wider range of PD symptomology and would maximize the ability to identify relationships between functional alterations and clinical status. Further, effects of disease progression would be better investigated using a longitudinal study paradigm. While such a broader approach was outside the scope of this particular study, the SSM-PLSC framework and the inclusion of age as a variable together with combining grouped data in multiple ways allowed us to provide important clinical context to observed metabolic disruptions and identify similarities in metabolic changes occurring as a function of age and disease. Including additional variables that track other aspects of PD symptomatology and including more diverse PD populations could allow PLSC to identify secondary, spatially distinct imaging biomarkers for these other aspects of disease. Specifically, the framework could be adapted to (i) incorporate non-motor symptoms into the suite of clinical variables (e.g., cognitive impairment, autonomic dysfunction, depression, apathy); (ii) including prodromal cohorts as separate groups (e.g., RBD, LRRK2 carriers); (iii) monitor disease-modifying therapies by separately grouping pre- and post-invention data (e.g., deep brain stimulation, novel drugs, lifestyle interventions). Furthermore, the framework presented can also be applied to other disease subtypes and/or neurodegenerative disorders.

Random permutation was employed to assess the generalizability of our derived patterns. The gold standard for pattern validation remains replicating the pattern with an independent cohort. Unlike SSM-PCA—which only requires an imaging dataset from an independent cohort—SSM-PLSC would require imaging data *and* an identical suite of clinical data from an independent cohort for external validation. To the best of our knowledge, no publicly available FDG datasets paired with clinical variables identical to those in our study exist. However, future SSM-PLSC studies could acquire FDG data and an expanded suite of clinical variables (such as those listed above), which would both enhance our present analysis and serve to validate our findings.

And lastly, while our work has used SSM-PLSC to provide enhanced clinical context to the metabolic changes in PD, the method is still ultimately limited to correlational rather than causal inferences. As a result, at this stage the biological interpretations of the identified patterns remain speculative and more targeted studies are required to further elucidate causal effects.

## Supplemental Material

sj-docx-1-pkn-10.1177_1877718X251394778 - Supplemental material for Novel insights into relationships between metabolic covariance patterns of FDG-PET data and clinical status in Parkinson's disease using partial least squares correlation analysis Supplemental material, sj-docx-1-pkn-10.1177_1877718X251394778 for Novel insights into relationships between metabolic covariance patterns of FDG-PET data and clinical status in Parkinson's disease using partial least squares correlation analysis by Connor WJ Bevington, Sahib Dhaliwal, Jessamyn McKenzie, A Jon Stoessl and Vesna Sossi in Journal of Parkinson's Disease
