# Supplementary material for: Novel insights into relationships between metabolic covariance patterns of FDG-PET data and clinical status in Parkinson's disease using partial least squares correlation analysis
Source: J Parkinsons Dis. 2025 Nov 11;16(1):72–86. doi: 10.1177/1877718X251394778 (PMC13347561; doi:10.1177/1877718X251394778)
Supplement: sj-docx-1-pkn-10.1177_1877718X251394778 - Supplemental material for Novel insights into relationships between metabolic covariance patterns of FDG-PET data and clinical status in Parkinson's disease using partial least squares correlation analysis [file sj-docx-1-pkn-10.1177_1877718X251394778.docx]

**SSM-PLSC: a more detailed mathematical description**

*Scaled Subprofile Modeling processing: computing the Subject Residual Profile*

Scaled Subprofile Modeling Principal Component Analysis (SSM-PCA) has been used for analyzing spatial covariance in PET and MRI datasets. In SSM, the imaging data are first log-transformed to reduce unimportant and potentially confounding global scaling factors into additive components. For instance, in the calculation of Standard Uptake Values with a reference region (SUVR), the choice of reference region may introduce variability unrelated to disease into the dataset. For the $i$th subject, any scaling factor $C_{i}$ is then removed by demeaning their log-transformed data by their global mean rate, $GMR_{i}$ (i.e., the mean over all brain regions/voxels):

$\log\left( \frac{I_{ij}}{C_{i}} \right)-\mathrm{mean}_{j}\left[ \log\left( \frac{I_{ij}}{C_{i}} \right) \right]=\log\left( I_{ij} \right)-\log\left( C_{i} \right)-\mathrm{mean}_{j}\left[ \log\left( I_{ij} \right)-\log\left( C_{i} \right) \right]$ $=\log\left( I_{ij} \right)-\log\left( C_{i} \right)+\log\left( C_{i} \right)-\mathrm{mean}_{j}\left[ \log\left( I_{ij} \right) \right]$ $=\log\left( I_{ij} \right)-\mathrm{mean}_{j}\left[ \log\left( I_{ij} \right) \right]$ $=\log\left( I_{ij} \right)-GMR_{i}$ (S1)

where $I_{ij}$ are the imaging data for the $i$th subject and $j$th region/voxel before applying the scaling factor. This subject-specific centring is important when there is high covariance across subjects, which is common in brain imaging: subjects within-group will have large similarities in brain function.

SSM then computes a group mean profile $GMP_{j}$ by taking the mean of $\log\left( I_{ij} \right)-GMR_{i}$ across subjects. $GMP_{j}$ is then subtracted from the subject-centred data to form the finalized SSM-processed data matrix, termed a Subject Residual Profile (SRP):

${SRP}_{ij}=\log\left( I_{ij} \right)-GMR_{i}-GMP_{j}$ (S2)

This second centring step is important where there is high covariance across regions/voxels, which is relevant for limited resolution imaging data and given the high degree of connectivity across brain regions. The doubly-centred data can thus be viewed as a residual profile from the average behaviour across subjects and regions, thus better highlighting disease-induced alterations. In the use of CMR_glu_ images in the present work [equation (1) of the main text], the subject-specific scaling of $C_{glu}$ and the common scaling of $LC$ are removed by SSM such that that residual profile only contains variance from the spatial distribution of $K_{i}$.

*Combining SSM with Partial Least Squares Correlation analysis (SSM-PLSC)*

PLSC provides a framework to explore the relationships between brain function and the clinical presentation of disease. Unlike applying PCA to the imaging data, PLSC applies Singular Value Decomposition (SVD) to a correlation matrix between external variables, i.e., clinical variables in the present context, and imaging data. The resulting decomposition identifies sets of spatial patterns and paired sets of clinical patterns (termed saliences in the PLSC literature). The projection of a given pattern pair onto their respective original datasets indicates the subject-specific expression of the spatial and clinical patterns (termed latent variables in the PLSC literature) that have maximal covariance; see Fig. 1 of the main text for a graphical illustration. As a result, PLSC uses the multivariate associations between the clinical variables and imaging data to reveal how functional brain alterations (described by the spatial patterns) are modulated by subjects’ clinical states (described by the patterns of clinical variables).

In combining SSM and PLSC, SRPs are first computed from the regional CMR_glu_ data as detailed above. PLSC pre-processing consists of z-scoring the imaging and clinical data across subjects for each region (for imaging data) or clinical variable (for clinical data). Since SRPs are already zero-centred across subjects, z-scoring after SSM processing additionally results in each regional distribution having a standard deviation of one. This allows one to form imaging data matrices ($\boldsymbol{X}$; subjects-by-ROIs) for the different grouping conditions: (i) PD and HC combined ($\boldsymbol{X}_{\boldsymbol{PD+HC}}$) and (ii) separate data matrices for PD ($\boldsymbol{X}_{\boldsymbol{PD}}$) and HC ($\boldsymbol{X}_{\boldsymbol{HC}}$). Separate data matrices for the clinical variables ($\boldsymbol{Y}$; subjects-by-variables) are formed and z-scored across subjects for each grouping condition, thus forming $\boldsymbol{Y}_{\boldsymbol{PD+HC}}$, $\boldsymbol{Y}_{\boldsymbol{PD}}$, and $\boldsymbol{Y}_{\boldsymbol{HC}}$.

By dint of the z-score normalization, a variables-by-ROI correlation matrix is formed as:

$\boldsymbol{R=}\boldsymbol{Y}^{\boldsymbol{T}}\boldsymbol{X}$. (S3)

PLSC then applies SVD to $\boldsymbol{R}$:

$\boldsymbol{R=U}\boldsymbol{\Sigma}\boldsymbol{V}^{\boldsymbol{T}}$ (S4)

thus identifying spatial patterns contained in the columns of $\boldsymbol{V}$ and clinical patterns in the columns of $\boldsymbol{U}$, which, when combined with the singular values in the diagonal matrix $\boldsymbol{\Sigma}$, reconstruct the original correlation matrix. Subject-specific expression for each pattern type can be computed by projecting the patterns back onto the original data matrices:

$\boldsymbol{S}_{\boldsymbol{X}}\boldsymbol{=XV}$ (S5)

$\boldsymbol{S}_{\boldsymbol{Y}}\boldsymbol{=YU}.$ (S6)

The $k$th column of $\boldsymbol{S}_{\boldsymbol{X}}$, i.e. $s_{X,k}$, contains the expression of the spatial pattern contained in the $k$th column of $\boldsymbol{V}$ and correspondingly $s_{Y,k}$ contains the expression of the clinical pattern contained in the $k$th column of $\boldsymbol{Y}$. PLSC is optimized such that $cov(s_{X,k},s_{Y,k'})$ is maximized for $k=k^{'}=1$, then the remaining covariance in the data is maximized for $k=k^{'}=2$, and so on. Furthermore, $cov\left( s_{X,k},s_{Y,k^{'}} \right)=0$ for $k\neq k'$, meaning the subject expressions from the $k$th pattern pair (i.e., the $k$th component) are uncorrelated to those from all other components. The amount of covariance accounted for by the $k$th component is determined using the singular values:

$CVAF_{k}=100\%\times\frac{\delta_{k}^{2}}{\sum_{k} \delta_{k}^{2}}$ (S7)

where $\delta_{k}$ is the $k$th diagonal element of $\boldsymbol{\Sigma}$.

SSM-PLSC is applied in three ways in the main text, yielding patterns with unique targets:

- **Metabolism differences in PD vs. HC:** SVD applied to $\boldsymbol{R}_{\boldsymbol{PD+HC}}\boldsymbol{=}\left( \boldsymbol{Y}_{\boldsymbol{PD+HC}} \right)^{\boldsymbol{T}}\boldsymbol{X}_{\boldsymbol{PD+HC}}$;
- **Patterns** **that track metabolism heterogeneity within PD:** SVD applied to $\boldsymbol{R}_{\boldsymbol{PD}}\boldsymbol{=}\left( \boldsymbol{Y}_{\boldsymbol{PD}} \right)^{\boldsymbol{T}}\boldsymbol{X}_{\boldsymbol{PD}}$;
- **C****ommon metabolism patterns that are expressed differently in PD and HC:** SVD applied to $\boldsymbol{R}=\left[ \begin{matrix} \boldsymbol{R}_{\boldsymbol{HC}} \\ \boldsymbol{R}_{\boldsymbol{PD}} \end{matrix} \right]=\left[ \begin{matrix} \left( \boldsymbol{Y}_{\boldsymbol{HC}} \right)^{\boldsymbol{T}}\boldsymbol{X}_{\boldsymbol{HC}} \\ \left( \boldsymbol{Y}_{\boldsymbol{PD}} \right)^{\boldsymbol{T}}\boldsymbol{X}_{\boldsymbol{PD}} \end{matrix} \right]$ reveals common spatial patterns to HC and PD with potentially unique intragroup relationships to the clinical metrics.

**Table of ROIs used for regional analysis**

All ROIs are taken from Freesurfer segmentations of each subject’s T1 image in their native space, unless otherwise specified.

| **Name** | **Description** | **Notes** |
| --- | --- | --- |
| L WM | Left cerebral white matter |  |
| L cer WM | Left cerebellar white matter |  |
| L cer GM | Left cerebellar grey matter |  |
| L thalamus | Left thalamus |  |
| L caudate | Left caudate |  |
| L putamen 1 | Left anterior putamen | Subsegmented from the Freesurfer putamen ROI along the anterior–posterior axis |
| L putamen 2 | Left middle putamen |  |
| L putamen 3 | Left posterior putamen |  |
| L pallidum | Left globus pallidus |  |
| L hippocampus | Left hippocampus |  |
| L amygdala | Left amygdala |  |
| L vent. striatum | Left ventral striatum |  |
| L substantia nigra | Left substantia nigra | Hand-drawn |
| R WM | Right cerebral white matter |  |
| R cer WM | Right cerebellar white matter |  |
| R cer GM | Right cerebellar grey matter |  |
| R thalamus | Right thalamus |  |
| R caudate | Right caudate |  |
| R putamen 1 | Right anterior putamen | Subsegmented from the Freesurfer putamen ROI along the anterior–posterior axis |
| R putamen 2 | Right middle putamen |  |
| R putamen 3 | Right posterior putamen |  |
| R pallidum | Right globus pallidus |  |
| R hippocampus | Right hippocampus |  |
| R amygdala | Right amygdala |  |
| R vent. striatum | Right ventral striatum |  |
| R substantia nigra | Right substantia nigra | Hand-drawn |
| Midbrain | Midbrain | Hand-drawn |
| Pons | Pons | Hand-drawn |
| Medulla | Medulla | Hand-drawn |
| L bankssts | Left banks of the superior temporal sulcus |  |
| L cau. ACC | Left caudal anterior cingulate cortex |  |
| L cau. mid. frontal | Left caudal middle frontal gyrus |  |
| L cuneus | Left cuneus |  |
| L entorhinal | Left entorhinal cortex |  |
| L fusiform | Left fusiform gyrus |  |
| L inf. parietal | Left inferior parietal cortex |  |
| L inf. temporal | Left inferior temporal cortex |  |
| L isthmus cingulate | Left isthmus cingulate cortex |  |
| L lat. occipital | Left lateral occipital cortex |  |
| L lat. orbitofrontal | Left lateral orbitofrontal cortex |  |
| L lingual | Left lingual gyrus |  |
| L med. orbitofrontal | Left medial orbitofrontal cortex |  |
| L mid. temporal | Left middle temporal gyrus |  |
| L parahippocampal | Left parahippocampal gyrus |  |
| L paracentral | Left paracentral lobule |  |
| L pars opercularis | Left pars opercularis |  |
| L pars orbitalis | Left pars orbitalis |  |
| L pars triangularis | Left pars triangularis |  |
| L pericalcarine | Left pericalcarine gyrus |  |
| L postcentral | Left postcentral gyrus |  |
| L PCC | Left posterior cingulate cortex |  |
| L precentral | Left precentral gyrus |  |
| L precuneus | Left precuneus |  |
| L rost. ACC | Left rostral anterior cingulate cortex |  |
| L rost. mid. frontal | Left rostral middle frontal gyrus |  |
| L sup. frontal | Left superior frontal gyrus |  |
| L sup. parietal | Left superior parietal lobule |  |
| L sup. temporal | Left superior temporal gyrus |  |
| L supramarginal | Left supramarginal gyrus |  |
| L front. pole | Left frontal pole |  |
| L temp. pole | Left temporal pole |  |
| L trans. temporal | Left transverse temporal gyrus |  |
| L insula | Left insular cortex |  |
| R bankssts | Right banks of the superior temporal sulcus |  |
| R cau. ACC | Right caudal anterior cingulate cortex |  |
| R cau. mid. frontal | Right caudal middle frontal gyrus |  |
| R cuneus | Right cuneus |  |
| R entorhinal | Right entorhinal cortex |  |
| R fusiform | Right fusiform gyrus |  |
| R inf. parietal | Right inferior parietal cortex |  |
| R inf. temporal | Right inferior temporal cortex |  |
| R isthmus cingulate | Right isthmus cingulate cortex |  |
| R lat. occipital | Right lateral occipital cortex |  |
| R lat. orbitofrontal | Right lateral orbitofrontal cortex |  |
| R lingual | Right lingual gyrus |  |
| R med. orbitofrontal | Right medial orbitofrontal cortex |  |
| R mid. temporal | Right middle temporal gyrus |  |
| R parahippocampal | Right parahippocampal gyrus |  |
| R paracentral | Right paracentral lobule |  |
| R pars opercularis | Right pars opercularis |  |
| R pars orbitalis | Right pars orbitalis |  |
| R pars triangularis | Right pars triangularis |  |
| R pericalcarine | Right pericalcarine gyrus |  |
| R postcentral | Right postcentral gyrus |  |
| R PCC | Right posterior cingulate cortex |  |
| R precentral | Right precentral gyrus |  |
| R precuneus | Right precuneus |  |
| R rost. ACC | Right rostral anterior cingulate cortex |  |
| R rost. mid. frontal | Right rostral middle frontal gyrus |  |
| R sup. frontal | Right superior frontal gyrus |  |
| R sup. parietal | Right superior parietal lobule |  |
| R sup. temporal | Right superior temporal gyrus |  |
| R supramarginal | Right supramarginal gyrus |  |
| R front. pole | Right frontal pole |  |
| R temp. pole | Right temporal pole |  |
| R trans. temporal | Right transverse temporal gyrus |  |
| R insula | Right insular cortex |  |

**Second SSM-PLSC component derived from combined HC and PD data**

SSM-PLSC identifies a second significant component from combined HC and PD data with a spatial pattern characterized by relative hypermetabolism primarily in bilateral cerebellar white matter, brain stem substructures, left globus pallidus, and inferior temporal cortex, plus relative hypometabolism in pre/postcentral gyrus, frontal regions, and insula (Fig. S1a). This pattern has a positive relationship with age (ρ=0.63, p<10^-5^) and a negative relationship with RBDSQ (ρ=-0.42, p<0.01), the latter still significant when analyzing PD-only subject scores (ρ=-0.48, p<0.05). No relationship is observed between disease duration/progression metrics, suggesting this pattern may be more related to metabolic signatures of the aging process.


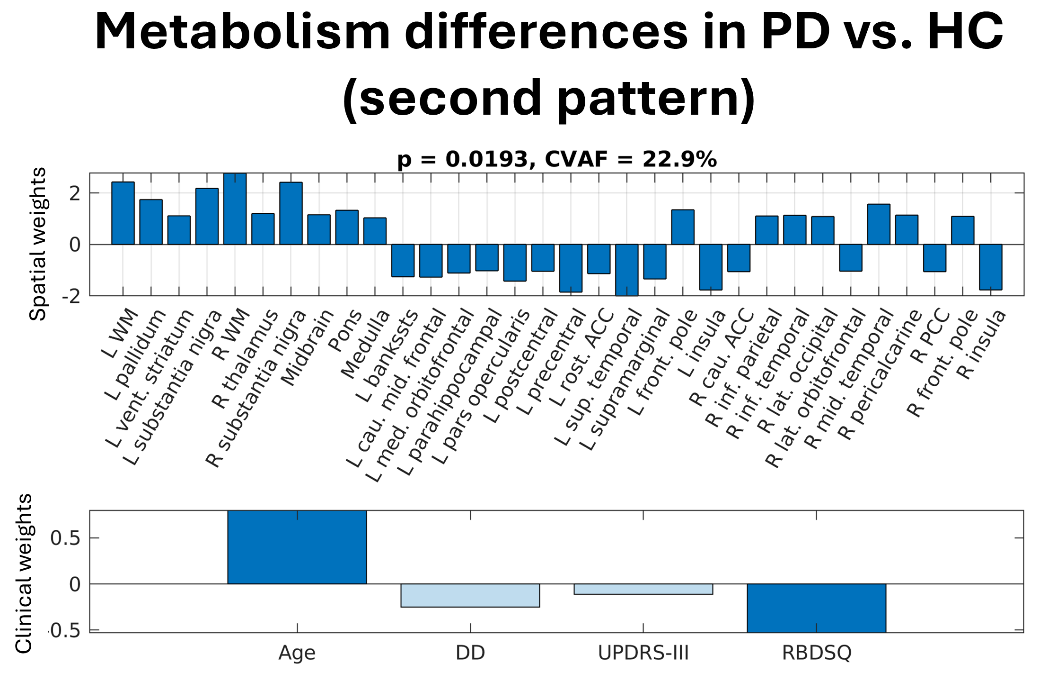


**Fig. S1.** The second spatial pattern (top pane) and clinical pattern (bottom pane) derived from combined HC and PD data, after accounting for the covariance of the first component (Fig. 2a of the main text). CVAF = percentage covariance accounted for by the pattern set; DD = disease duration; UPDRS-III = Unified Parkinson’s Disease Rating Scale Part III; RBDSQ = REM sleep Behaviour Disorder Screening Questionnaire.

In terms of interpretation, it should be noted that this second component is derived *after accounting for covariance in the first component* (Fig. 2a of the main text); since PD vs. HC covariance is primarily accounted for in the first component, any remaining age-related covariance common to PD and HC and is likely to be capture in a secondary component. As demonstrated in Fig. S2, the linear combination of these first two components yields a combined spatial pattern whose weights strongly correlate with the weights of the PD and HC spatial pattern, (Fig. 5a of the main text).


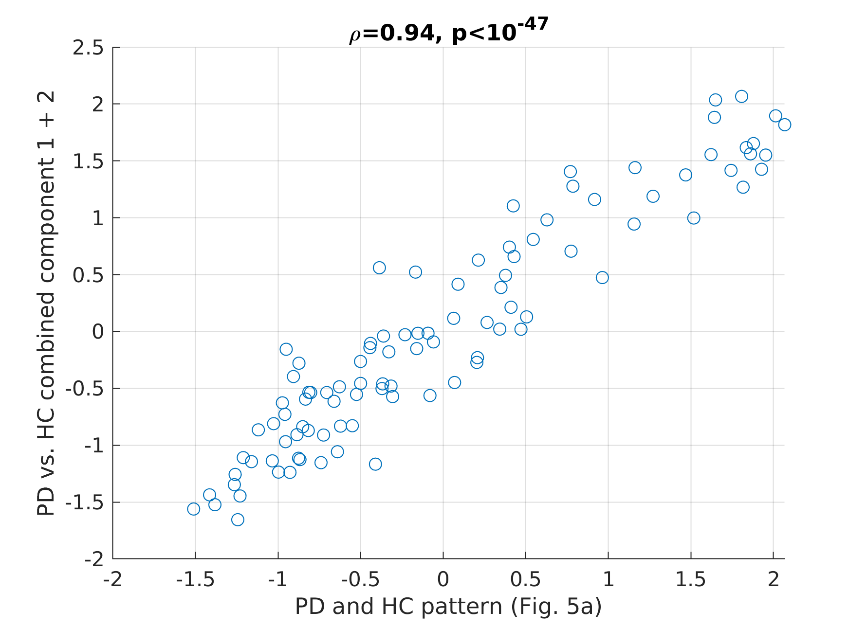


**Fig. S2.** Comparing the spatial weights after combining components 1 and 2 of the PD vs. HC analysis (Fig. 2a of the main text and Supplementary Fig. 1, respectively) with the corresponding spatial weights of the PD and HC pattern (Fig. 5 of the main text).
